# Prevalence of Sexual Violence Against Children and Use of Social Services — Seven Countries, 2007–2013

**Published:** 2015-06-05

**Authors:** Steven A. Sumner, James A. Mercy, Janet Saul, Nozipho Motsa-Nzuza, Gideon Kwesigabo, Robert Buluma, Louis H. Marcelin, Hang Lina, Mary Shawa, Michele Moloney-Kitts, Theresa Kilbane, Clara Sommarin, Daniela P. Ligiero, Kathryn Brookmeyer, Laura Chiang, Veronica Lea, Juliette Lee, Howard Kress, Susan D. Hillis

**Affiliations:** 1 Division of Violence Prevention, National Center for Injury Prevention and Control, CDC; 2 Epidemic Intelligence Service, CDC; 3 Division of Global HIV/AIDS, Center for Global Health, CDC; 4 Ministry of Health, Swaziland; 5 School of Public Health and Social Sciences, Muhimbili University of Health and Allied Sciences, Tanzania; 6 Kenya National Bureau of Statistics; 7 Interuniversity Institute for Research and Development, Haiti; 8 National Institute of Statistics, Cambodia Ministry of Planning; 9 Malawi Ministry of Gender, Children and Social Welfare; 10 Together for Girls; 11 Child Protection Programme Division, United Nations Children’s Fund; 12 Office of the Global AIDS Coordinator, U.S. Department of State; 13 Division of STD Prevention, National Center for HIV/AIDS, Viral Hepatitis, STD, and TB Prevention

Sexual violence against children erodes the strong foundation that children require for leading healthy and productive lives. Globally, studies show that exposure to violence during childhood can increase vulnerability to a broad range of mental and physical health problems, ranging from depression and unwanted pregnancy to cardiovascular disease, diabetes, and sexually transmitted diseases, including human immunodeficiency virus (HIV) (1,2). Despite this, in many countries, the extent of sexual violence against children is unknown; estimates are needed to stimulate prevention and response efforts and to monitor progress. Consequently, CDC, as a member of the global public-private partnership known as Together for Girls,* collaborated with Cambodia, Haiti, Kenya, Malawi, Swaziland, Tanzania, and Zimbabwe to conduct national household surveys of children and youth aged 13–24 years to measure the extent of violence against children. The lifetime prevalence of experiencing any form of sexual violence in childhood ranged from 4.4% among females in Cambodia to 37.6% among females in Swaziland, with prevalence in most countries greater than 25.0%. In most countries surveyed, the proportion of victims that received services, including health and child protective services, was ≤10.0%. Both prevention and response strategies for sexual violence are needed.

During 2007–2013, CDC and UNICEF, in partnership with host country governments, communities, and academic institutions developed and administered Violence Against Children Surveys (VACS) in seven countries. The first VACS were administered in Swaziland in 2007; most recently, VACS were administered in Malawi in 2013. Protocols were approved by host country and CDC institutional review boards. VACS are a multistage cluster survey with national coverage, administered by host country survey workers (trained by CDC and local partners) via household, face-to-face interviews. Surveys are initiated at the request of host-country governments. Informed consent/assent is obtained from all participants, special safeguards are incorporated for confidentiality, all participants receive a referral list of available services, and any victims desiring aid are referred for social services. This report focuses on lifetime childhood sexual violence (before age 18 years) among male and female respondents aged 18–24 years. Sexual violence included unwanted touching, unwanted attempted sex, pressured/coerced sex, and forced sex. Sex was specifically defined as vaginal/anal penetration by the penis, hands, fingers, mouth, or objects, or oral penetration by the penis except in Swaziland (penetration of vagina/anus by penis only) and Malawi (oral, vaginal, or anal sex or vaginal/anal object insertion).

Patterns in the prevalence of any form of childhood sexual violence differed by country (Figure). Swaziland had high reported prevalence of sexual violence among females (37.6%). Reported sexual violence among females in Zimbabwe also was high (32.5%), yet Zimbabwe had a considerably lower reported prevalence of sexual violence against males (8.9%). Haiti had high prevalence rates for both males (21.2%) and females (25.7%). Cambodia reported the lowest rates for both females (4.4%) and males (5.6%).

Among respondents who reported childhood sexual violence, the proportion who also reported receiving services, including health care, legal/security aid, or counseling support, was low for both males and females (Table 1). Swaziland had the largest proportion (24.0%) of females receiving services. In a few countries, data were readily available on the proportion of children who sought services in addition to the percentage who received services. In Malawi, 9.6% of female and 5.9% of male victims sought services. In Kenya, 6.8% of females and 2.1% of males attempted to seek services. Finally, in Tanzania, 16.2% of female and 10.8% of male victims sought services. Among all victims in these countries, the proportion receiving services was no higher than 11.7% (female victims in Tanzania).

Completed acts of unwanted sex (i.e., pressured or forced penetrative sex acts) generally were higher among females than males (Table 2). Approximately 17.5% of females in Swaziland reported experiencing an episode of unwanted, completed sex. The lifetime childhood prevalence of unwanted, completed sex also was high among females in Zimbabwe (13.5%), Kenya (11.8%), and Haiti (9.0%).

## Discussion

Rates of sexual violence against children are high in many of the countries studied. In most of the countries, >25% of females and >10% of males reported experiencing childhood sexual violence. Furthermore, in approximately 50% of the countries, >10% of females reported experiencing completed, unwanted, penetrative sex. In spite of this, few children sought services after the abuse, and not all who sought services received them.

The findings in this report present the results of a broad international collaboration to address sexual violence; such violence is both a fundamental violation of children’s rights and a major risk factor for a wide range of illnesses. The first step in addressing violence against children has been to determine comparable international estimates of the magnitude of the problem. Most previous studies have had some important limitations as they mainly focused on school-based populations; however, these children might not be fully representative of a nation’s youth (3).

Accurately quantifying and then addressing sexual violence is integral to achieving several major global health aims, including HIV prevention. The research on child sexual violence presented in this report was conducted in several countries with generalized HIV epidemics that are partnered with the President’s Emergency Plan for AIDS Relief. Studies have linked experiencing violence to later high-risk sexual activities, including further sexual exploitation, multiple sex partners, experience or perpetration of rape, unwanted pregnancy, and HIV acquisition (1,4,5). It is essential to target sexual violence as a component of HIV and other disease prevention strategies.

Beyond HIV and other sexually transmitted diseases, experiencing violence as a child has been linked with several noncommunicable diseases including heart disease, cancer, diabetes, and tobacco, alcohol, and drug addiction, among others (2). Internationally, recognition of the importance of addressing noncommunicable diseases in low- and middle-income countries is growing (6). Experiencing trauma as a child can contribute to biologic changes, such as altered hormonal responses as well as mental illness, such as depression, or other psychological changes like poor social relations and low self-esteem, all of which elevate risk for developing chronic diseases (7). Primary prevention of sexual violence can help avert some of these long-lasting and often treatment-resistant consequences.

Despite myriad adverse effects of sexual violence, in this study, most persons who reported experiencing it during childhood did not receive services for their abuse. From a limited number of countries, it appeared that few children even seek services, possibly because they are not aware of service availability, services may not be readily available, or stigma may exist. Although the control of and response to violence traditionally has been seen as the responsibility of law enforcement and social welfare, health sectors can integrate violence prevention and care into routine programmatic activities, building clear links to social services to achieve maximal benefit for various health measures. For example, improved identification of those experiencing violence and the subsequent delivery of counseling, emergency housing, or legal/protection assistance might aid victims. The results of this study provide nationally representative estimates by which countries can measure their progress in reducing childhood violence.

What is already known on this topic?

Preventing sexual violence against children is essential. Childhood victims of sexual violence are at a significantly increased risk for numerous adverse health outcomes ranging from HIV acquisition to poor mental health and chronic disease development.

What is added by this report?

The prevalence of childhood sexual violence among females was ≥25% in five of seven countries surveyed. Among males in four of six countries, there was a prevalence of childhood sexual violence ≥11%. Despite the prevalence, the proportion of victims receiving service was ≤10% in most countries surveyed.

What are the implications for public health practice?

Sexual violence against children is common yet most children go unaided. Countries should work to assess, respond to, and prevent childhood sexual violence. THRIVES, a broad technical package, can help countries identify programs and strategies than can prevent and treat sexual violence.

Research on both the primary prevention of sexual violence and treatment of its consequences largely has focused on awareness-raising and educational interventions or therapy-based approaches (8,9). The prevention of sexual violence and the promotion of safe, stable, and nurturing relationships and environments for children need more research, as does the assessment of other social, structural/environmental, or clinical approaches. VACS are creating a foundation for such actions. As part of the arrangements to execute VACS, host countries develop response plans based on survey findings. Country-level responses have included legislation, school-based educational curriculum, media outreach, service provision strategies, and an increase in the workforce capacity of clinicians, police, social workers, and teachers, among others. Thus, beyond simply disseminating information, VACS have a built-in mechanism intended to raise awareness, catalyze action, and effect change. THRIVES is a technical package that can help countries in identifying programs and policies that are effective in preventing and responding to violence against children (Box).

The findings in this report are subject to at least four limitations. First, recall bias might be present, particularly for remote episodes of abuse. Second, limited disclosure might have occurred because of the sensitive nature of the subject. Third, children not residing in households (namely, street children) are not included. Lastly, data from some countries might no longer be representative of current levels of violence of services because of the year of data collection.

The Together for Girls public-private partnership, which includes technical expertise from the World Health Organization, the Joint United Nations Programme on HIV/AIDS, UNICEF, CDC, USAID, the Presidents Emergency Plan for AIDS Relief, and other partners, has identified a multifaceted strategy to begin to address sexual violence (10). The first objective is to gather accurate surveillance data. Subsequent important responses identified by the partnership include supporting government and civil society activities to prevent violence, strengthening local government response plans and institutional capacity, improving public awareness, ensuring the creation of appropriate national legislation, mobilizing and promoting community-based strategies, and improving access to and quality of services available to those experiencing violence (10). VACS, as a key tool of a broad, international collaboration demonstrates the feasibility of a multisectoral approach to the investigation and prevention of childhood violence.

BOX THRIVES,* strategies to help countries reduce violence against children — CDC, 2015

**Table:** 

THRIVES represents a select group of complementary strategies that reflect the best available evidence to help countries reduce violence against children. These strategies cross health, social services, education, finance, and justice sectors: Training in parenting Increase bonding and positive parent-child interactions, and reduce harsh and violent parenting practices. Household economic strengthening Decrease violence through use of cash transfers and savings/loan programs together with gender norms and/or equity training. Reduced violence through protective policies Promote laws or regulations (e.g., prohibit sexual abuse/exploitation/violent punishment; regulate alcohol). Improved services Support counseling services that are effective in reducing trauma-related symptoms. Values and norms that protect children Change harmful attitudes and beliefs through interventions (e.g., bystander programs, campaigns, small group/community mobilization programs). Education and life skills Increase school enrollment/attendance and build life skills with programs that empower girls, prevent dating violence, and prevent rape. Surveillance and evaluation Monitor and evaluate periodically to manage and improve THRIVES-based programs and policies after implementation.

## Figures and Tables

**FIGURE:**
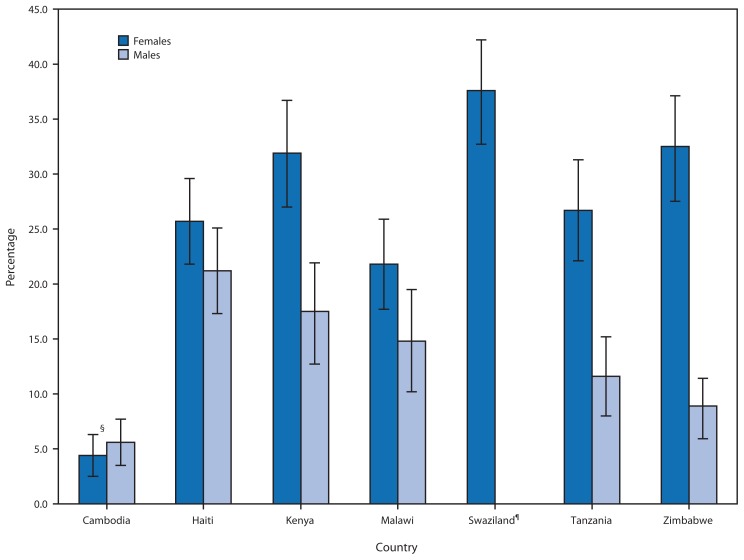
Lifetime prevalence of experiencing any form of sexual violence* before age 18 years among respondents aged 18–24 years, by country^†^ * Any sexual violence includes unwanted sexual touching, unwanted attempted sex, pressured/coerced sex, or forced sex. ^†^ All numbers represent weighted percentages. ^§^ 95% confidence interval. ^¶^ In Swaziland, only females were surveyed.

**TABLE 1:** Percentage of persons aged 18–24 years who received services among those who experienced any form of sexual violence when aged <18 years, by country — seven countries, 2007–2013

	Females		Males	
		
Country	%	(95% CI)	%	(95% CI)
Cambodia	NA	NA	NA	NA
Haiti*	10.0	(1.9–18.1)	6.6	(1.9–11.2)
Kenya	3.4	(0.0–7.0)	0.4	(0.0–1.3)
Malawi†	9.0	(0.0–22.8)	5.9	(0.0–11.7)
Swaziland	24.0	(18.0–30.1)	—§	—§
Tanzania	11.7	(4.6–18.9)	4.9	(0.0–14.1)
Zimbabwe	2.7	(0.4–5.0)	2.4	(0.0–5.5)

**Abbreviations:** CI = confidence interval; NA = data not available.

* Defined as talking to or receiving services from a professional health care worker, legal personnel, security or police service, or professional counselor.

† Defined as receiving help from a hospital, clinic, police station, helpline, social welfare, or legal office.

§ In Swaziland, only females were surveyed.

**TABLE 2:** Lifetime prevalence of experiencing unwanted completed sex* before age 18 years among survey respondents aged 18–24 years, by country† — seven countries, 2007–2013

	Females§		Males§	
		
Country	%	(95% CI)	%	(95% CI)
Cambodia	1.5	(0.3–2.8)	0.2	(0.0–0.5)
Haiti	9.0	(6.3–11.8)	7.6	(5.1–10.1)
Kenya	11.8	(8.5–15.2)	3.6	(1.6–5.6)
Malawi	6.7	(3.7–9.8)	1.9	(0.3–3.6)
Swaziland	17.5	(13.8–21.2)	—¶	—¶
Tanzania	6.1	(3.3–8.8)	2.7	(0.8–4.7)
Zimbabwe	13.5	(10.3–16.6)	1.8	(0.8–2.8)

**Abbreviation:** CI = confidence interval.

* Unwanted completed sex includes pressured/coerced sex and forced sex.

† Survey years, total survey respondents, and response rates detailed in country reports available at http://www.cdc.gov/violenceprevention/vacs/vacs-reports.html.

§ Numbers represent weighted percentages.

¶ In Swaziland, only females were surveyed.

## Footnotes

* Other partners in Together for Girls include host country governments, UNICEF, the President’s Emergency Plan for AIDS Relief (PEPFAR), and other organizations. More information is available at http://www.togetherforgirls.org.

* Additional information available at http://www.cdc.gov/violenceprevention/vacs/resources.html.
